# *In situ* gelling hydrogel loaded with berberine liposome for the treatment of biofilm-infected wounds

**DOI:** 10.3389/fbioe.2023.1189010

**Published:** 2023-05-31

**Authors:** Sipan Li, Yongan Wang, Siting Wang, Jianjun Xie, Tingming Fu, Shaoguang Li

**Affiliations:** ^1^ School of Pharmacy, Nanjing University of Chinese Medicine, Nanjing, China; ^2^ Microsurgery Department of Senior Department of Orthopedics, The Fourth Medical Center of PLA General Hospital, Beijing, China

**Keywords:** wound infection, biofilm, liposomes, berberine, temperature-sensitive gel, *Staphylococcus aureus*

## Abstract

**Background:** In recent years, the impact of bacterial biofilms on traumatic wounds and the means to combat them have become a major research topic in the field of medicine. The eradication of biofilms formed by bacterial infections in wounds has always been a huge challenge. Herein, we developed a hydrogel with the active ingredient berberine hydrochloride liposomes to disrupt the biofilm and thereby accelerate the healing of infected wounds in mice.

**Methods:** We determined the ability of berberine hydrochloride liposomes to eradicate the biofilm by means of studies such as crystalline violet staining, measuring the inhibition circle, and dilution coating plate method. Encouraged by the *in vitro* efficacy, we chose to coat the berberine hydrochloride liposomes on the Poloxamer range of *in-situ* thermosensitive hydrogels to allow fuller contact with the wound surface and sustained efficacy. Eventually, relevant pathological and immunological analyses were carried out on wound tissue from mice treated for 14 days.

**Results:** The final results show that the number of wound tissue biofilms decreases abruptly after treatment and that the various inflammatory factors in them are significantly reduced within a short period. In the meantime, the number of collagen fibers in the treated wound tissue, as well as the proteins involved in healing in the wound tissue, showed significant differences compared to the model group.

**Conclusion:** From the results, we found that berberine liposome gel can accelerate wound healing in *Staphylococcus aureus* infections by inhibiting the inflammatory response and promoting re-epithelialization as well as vascular regeneration. Our work exemplifies the efficacy of liposomal isolation of toxins. This innovative antimicrobial strategy opens up new perspectives for tackling drug resistance and fighting wound infections.

## 1 Introduction

It has been reported that more than 10 million people are infected by disease-causing bacteria each year, and death from bacterial infections is the second leading cause of death worldwide (Hu et al., 2017). Wounds caused by various natural and human factors are the main route of bacterial invasion, making wound infections account for 60%–80% of human bacterial infections. In our daily life, wound infections are often caused by delayed wound treatment or poor antibacterial properties of therapeutic drugs. Moreover, chronic wounds have become one of the major global health problems due to an aging population and the increasing prevalence of diseases such as diabetes and obesity. The main reason why infectious diseases are difficult to deal with was thought to be the bacterial resistance caused by the overuse of antibiotics (Turner et al., 2019; Huemer et al., 2020; LaPlante et al., 2022). However, recent studies have shown that another major reason for the failure of many wound infection treatments is the formation of biofilms, which are difficult for many antimicrobial agents to penetrate (Roy et al., 2020; Lin et al., 2021; Rubio-Canalejas et al., 2022).

**SCHEME 1:**
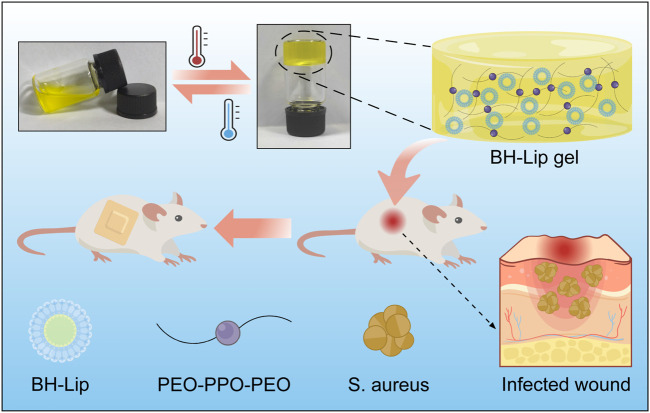
Schematic diagram of the formation of berberine liposomal thermosensitive *in situ* gel and its promotion of wound healing in *S. aureus* infections.

In nature, the vast majority of bacteria exist as a biofilm rather than as plankton (Sharahi et al., 2019). Bacterial communities tend to attach to the surfaces they come in contact with and encase themselves in self-produced extracellular polymeric substances (EPS), and the bacterial aggregates resulting from this community activity of bacteria are referred to as biofilms. The main component of the EPS is water and also includes lipids, polysaccharides, proteins, etc., which shapes a good habitat for the bacteria to communicate with the external environment and also protects them from the immune system (Zhou et al., 2018; Yan and Bassler, 2019; Jiang et al., 2021). In other words, biofilms provide a natural protective barrier for bacterial communities, and it is this protective effect of the biofilm on the bacteria that makes the eradication of the bacteria so tough. The ability of biofilms to protect themselves is as virulent as it is extremely difficult to completely remove them from the infected wound. Therefore, there is an urgent need to develop a new therapeutic strategy to combat biofilms on wounds. Scheme 1.

In the past decade, liposomes have become a hot research topic as drug carriers, since their lipid-like bilayer structure has good biocompatibility and the ability to encapsulate drugs (Chang and Yeh, 2012; Forier et al., 2014; Scriboni et al., 2019; Kluzek et al., 2022). We recently combined artificially designed liposomes with berberine to enable the berberine liposomes (BH-Lip) to target biofilms and promote drug release, which has played a very good role in the eradication of *Staphylococcus aureus* (*S. aureus*) biofilms (Xie et al., 2022). Moreover, it was found that liposomes can not only encapsulate drugs but also can sequester bacterial toxins. The synthetic liposomes with a high concentration of cholesterol (66 mol% cholesterol) as a decoy effectively bind protein toxins secreted by many bacterial pathogens, thus protecting the host cells from the toxins (Henry et al., 2015). The outstanding antibacterial and cytoprotective functions of berberine liposome provide the possibility for its application in infected wounds.

As a typical biocompatible wound dressing, hydrogels exhibit superior characteristics similar to the natural extracellular matrix and has been extensively applied in the treatment of wound infections (Liu et al., 2020), inhibition of post-surgery tumor recurrence (Li et al., 2022a), prevention of postoperative adhesion (Zhou et al., 2022). Compared with conventional preformed hydrogels, temperature-sensitive *in situ* gelling hydrogels have the property of undergoing a self-solution to gel phase transition with temperature changes at the drug application site and are considered promising materials for wound dressings (Yan et al., 2019; Zhang et al., 2019; Park et al., 2022). Poloxamer series gels are widely recognized for their good flowability, short gelling time, and excellent drug compatibility (Wang et al., 2019; Yang et al., 2020; Liang et al., 2021; Zhang et al., 2021). Recently, Li et al., (2022b) developed an injectable *in situ* gel for the peri-tumoural administration of primary and metastatic tumours by using Pluronic F127. Peng et al. (2022) designed a Poloxamer 407 and hyaluronic acid thermosensitive hydrogel encapsulated Ginsenoside Rg3 to promote skin wound healing. In this study, *in situ* gelling hydrogel loaded with berberine hydrochloride liposome (BH-Lip gel) was used to treat skin wounds of mice infected with *S. aureus*. According to the results of *in vivo* experiments, the hydrogel loaded with BH-Lip greatly eradicated the biofilm on the wound and promoted wound healing. This multifunctional thermosensitive hydrogel with simple preparation and low-cost design by us has a broad development prospect and is expected to be used clinically for the treatment of wound infection caused by bacteria.

## 2 Materials and methods

### 2.1 Materials

BH was purchased from Liangwei Biotechnology Co., Ltd. (Nanjing, China). HSPC, 1, 2-distearoyl-sn-glycerol-3-phosphoethanolamine-N-[methoxy (polyethylene glycol)-2000] (DSPE-PEG2000), and cholesterol were purchased from AVT (Shanghai) Pharmaceutical Tech Co., Ltd. *S. aureus* (ATCC25923), Poloxamer 407, and Poloxamer 188 were purchased from Solarbio Science Technology Co., Ltd. (Beijing, China). ELISA kits were purchased from Nanjing YIFEIXUE Biotechnology Co., Ltd. All other reagents were commercially purchased and were not further purified.

### 2.2 Preparation of blank liposome

The protocol for the preparation of liposomes was slightly modified from the previous study (Xie et al., 2022). The components of the liposomes were Hydrogenated Soybean Phosphatidylcholine (HSPC), cholesterol, and DSPE-PEG_2000_. Initially, HSPC, cholesterol, and DSPE-PEG_2000_ were dispersed in an eggplant flask containing chloroform at a molar ratio of 45:50:5 and gently shaken until completely dissolved. Then the organic solvent was removed in a rotary vacuum evaporator at 60°C. After the solvent was completely evaporated, the lipid film was formed. Then a citric acid buffer (50 × 10^−3^ M citric acid, 23.8 × 10^−3^ M sodium citrate) was added as a hydration medium to form a lipid suspension at a concentration of 5 mg/mL. Next, the liposomes were sonicated for 5 min in an ultrasonic cell disruption apparatus. Finally, the suspension was extruded through a polycarbonate membrane (Whatman plc, Buckinghamshire, United Kingdom) with a pore size of 0.2 μm for 6 times at a temperature higher than the lipid transition temperature (60°C), and then extruded through a polycarbonate membrane with a pore size of 0.1 μm for 11 times to obtain liposomes of uniform particle size. Determination of liposome size distribution using dynamic light scattering (Zetasizer Nano ZS90; Malvern Panalytic, Malvern, United Kingdom).

### 2.3 Preparation of antibiotic liposomes

The pH gradient method was used to prepare berberine liposomes. First, the blank liposome suspension obtained by the above method was mixed with berberine hydrochloride solution (1 mg/mL) at a volume ratio of 2:1. Then 595 × 10^−3^ mM NaHCO_3_ solution was slowly added to adjust the pH of the external phase to 7.0, and dilute the berberine hydrochloride concentration to 200 μg/mL with PBS. Finally, the mixed solution was incubated in a water bath at 60°C for 20 min and then subjected to rapid cooling, and the obtained BH-Lip solution was stored at 4°C. Liposomes containing ciprofloxacin or ceftazidime were prepared using the same method as BH-Lip, with the difference that ciprofloxacin hydrochloride aqueous solution or ceftazidime aqueous solution was used instead of berberine hydrochloride solution.

### 2.4 Antibacterial test process

The drug sensitivity test followed the standard method of CLSL (Clinical And Laboratory Standards Institute) with minor adjustments for the determination of minimum inhibitory concentration (MIC) using the micro broth dilution method (Nicolosi et al., 2015). Firstly, the concentration of *S. aureus* was adjusted to 10^6^ CFU/mL with TSB liquid medium (10^8^ CFU/mL at OD_600_ = 0.4 abs). and then 100 μL of the above-mentioned bacterial solution with a concentration of 10^6^ CFU/mL was added to 100 μL of BH-Lip solution which was serially diluted 2 times, and one column of the 96-well plate was used as the negative control (100 μL of bacterial solution mixed with 100 μL of TSB), and the other column was used as the blank control (diluted the drug solution with sterile PBS). After incubation at 37°C for 24 h, the absorbance at 600 nm was measured using an enzyme marker, and the MIC of the drug was determined by calculating the inhibition rate.

### 2.5 Determination of the inhibition circle

For the determination of the inhibition circle, the culture was diluted to 10^6^ CFU/mL, and 100 μL was pipetted onto TSA agar plates using a micropipette. Then 4 wells were punched on the agar plate with a hole puncher (6 mm diameter) and 50 μL of the solution was added dropwise to each well and incubate the agar plate at 37°C for 18–24 h to observe the range of the inhibition circle.

### 2.6 Determination of biofilm eradication effectiveness

The eradication rate of BH-Lip on the biofilm of *S. aureus* was determined by the crystalline violet staining method. Firstly, the bacterial concentration was adjusted to 10^6^ CFU/mL by TSB, and then the bacterial solution with proper concentration was added to columns 2 to 11 of the 96-well plate (the middle 6 wells in each column were added 200 μL of bacterial solution), and the remaining wells were sealed with 200 μL of sterile PBS, and then left at 37°C for incubated for 24 h to form biofilms. BH-Lip solution was diluted to 200, 100, 50, 25, and 12.5 μg/mL respectively, 200 μL of each concentration of BH-Lip was added to the well and incubated with biofilm at 37°C for 24 h. 200 μL of sterile PBS to the negative control group. After reaching the incubation time, the supernatant was aspirated and discarded, the biofilm was washed with sterile PBS several times. 200 μL of methanol was added to each well, and after fixation for 15 min, the residual methanol was allowed to evaporate for 30 min at room temperature. 200 μL of 0.1% crystalline violet was added to each well and incubated for 30 min, the crystalline violet solution was then aspirated and discarded, and the biofilm was washed several times with sterile PBS and dried at room temperature. 200 μL of 30% glacial acetic acid solution was added to each well to dissolve the crystalline violet, 100 μL of each well was aspirated and the absorbance was measured at 595 nm with a microplate reader to calculate the biofilm eradication rate. The Ceftazidime liposomes (CAZ-Lip) and Ciprofloxacin liposomes (CIP-Lip) eradication experiments for *S. aureus* biofilms were performed as described above.

### 2.7 Preparation of *in-situ* thermosensitive hydrogel

The *in-situ* thermosensitive hydrogels were prepared using a cold dissolution method (Khallaf et al., 2022). For the blank gel, a certain mass of Poloxamer 407 (P407) and Poloxamer 188 (P188) was dissolved in cold deionized water (4°C–8°C) to a concentration of 23% for P407 and 3% for P188, stirred continuously for 1 h in an ice water bath, and then placed under refrigeration at 4°C overnight to ensure complete dissolution. The BH gel (BH concentration of 200 μg/mL), BH-Lip gel (BH-Lip concentration of 200 μg/mL), and Lip gel (2 mg/mL) were prepared by mixing the blank gel with the corresponding substance (BH, BH-lip or blank Lip).

### 2.8 Measurement of gelation temperature and time

The gelling temperature was measured using the vial tilt method (Fathalla et al., 2017). A 2 mL sample was added to the vial, and the vial was put into a water bath where the water temperature was increased from 25°C to 45°C at a rate of 1°C/min. During this period, the temperature at which the sample changed from solution to gel was recorded (each gel was measured three times and the average was taken as the result). After the phase transition temperature of the gel was determined, 150 μL of the sample was pipetted onto a hot aluminum pan that maintains the phase transition temperature, and then the pan was tilted by 90° and the time for the gel to stop flowing was recorded in seconds (repeat the test three times, and take the average value).

### 2.9 *In vitro* antibacterial activity of thermosensitive hydrogel

The antibacterial performance of hydrogels was evaluated using the colony counting method. Firstly, 500 μL of hydrogel was aspirated in sterile centrifuge tubes, then 500 μL of 10^6^ CFU/mL of *S. aureus* solution was added sequentially and incubated at a constant temperature of 37°C for 24 h. Finally, 100 μL of the incubated suspension after 10^3^-fold gradient dilution were pipetted onto TSA agar plates and incubated at 37°C for 18–20 h to observe the growth of colonies. The inhibition of *S. aureus* by the gels at different periods was performed in 24-well plates. 500 μL of gel was pipetted with 500 μL of 10^6^ CFU/mL of bacterial solution in a 24-well plate (6 wells of each gel were taken as a parallel control), and a mixture of 500 μL of PBS solution and 500 μL of 10^6^ CFU/mL of bacterial solution was used as a control. Following, the 24-well plates were incubated in a thermostat (37°C) for 0 h, 6 h, 12 h, and 24 h, and then 4 wells were randomly selected for each group until the corresponding time. 10 μL of the mixed solution was added dropwise to the agar plates and incubated in a thermostat (37°C) for 18–24 h to observe the antimicrobial effect, and the remaining solution was used for absorbance measurement.

### 2.10 *In vivo* antibacterial effect and wound healing properties

*In vivo S. aureus* infection experiments were performed using male BALB/c mice (6–8 weeks). Mice were randomly divided into 6 groups, and two symmetrical circular wounds were cut on the back of each mouse except the blank group using a 6-mm diameter punch. Infective wounds were prepared by dropping 100 μL of *S. aureus* solution (10^7^ CFU/mL) into the wound. The day of infection was recorded as day 0, and the drug administration was started on the first day after infection (150 μL of saline as the control in the model group and 150 μL of gel in the other four groups) and continued for 13 days. During the treatment period, the body weight and wound area of each mouse were measured and recorded every other day.

On day 5, three mice in each group were randomly selected to be euthanized. A portion of wound tissue was homogenized and centrifuged at 5,000 × g at 4°C for 10 min, and the supernatant was collected for the determination of interleukin 1β (IL-1β), interleukin 6 (IL-6), and tumor necrosis factor-α (TNF-α). Some tissue homogenates were serially diluted 10^3^ folds with PBS, and 100 μL of the diluted homogenates were pipetted onto TSA agar to quantify bacterial colonies. Another portion of the tissue was fixed with *in situ* hybridization fixative and used for fluorescence *in situ* hybridization (FISH) experiments. On day 14 of treatment, all mice were euthanized and a portion of the wound tissue was taken for IL-1β, IL-6, and TNF-α, while the rest of the wound tissue was used for H&E and Masson’s staining and immunofluorescence for cytokeratin 14 (CK14), Vascular endothelial growth factor (VEGF) and protein F4/80.

### 2.11 Statistical analysis

All the data in this study were statistically analyzed by One-way ANOVA (intergroup comparisons) and Student’s t-test (intragroup comparisons). The values were presented as mean ± standard deviation (SD), *p* < 0.05 was considered statistically significant (*p < 0.05, **p, ***p < 0.01).

## 3 Results

### 3.1 Berberine-loaded liposomes successfully inhibited *S. aureus* and eradicated biofilm

**FIGURE 1:**
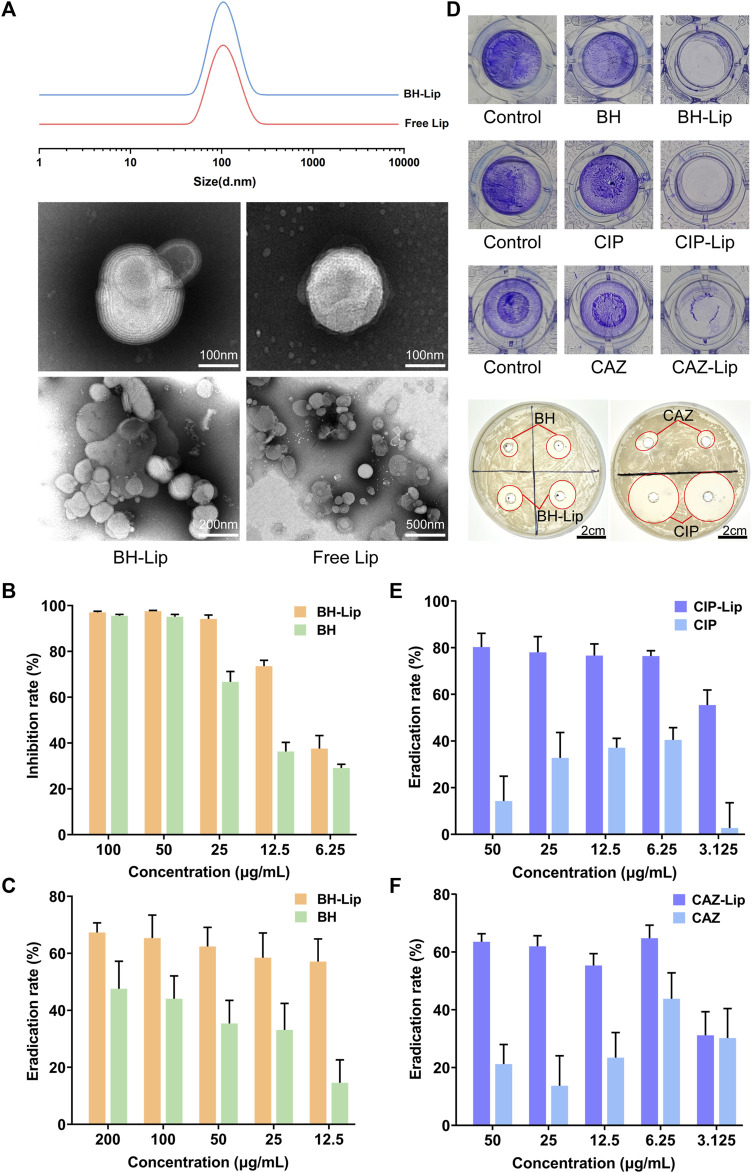
The particle size of BH-Lip and Free Lip and their TEM images **(A)**. Inhibition of planktonic *S. aureus* by BH-Lip. (*n* = 6/group) **(B)**. The eradication rate of BH-Lip (*n* = 5/group) **(C)**. Difference between the administered group and the control group after crystalline violet staining and the circle of inhibition of the different drugs **(D)**. The eradication rate of CIP-Lip (*n* = 5/group) **(E)**. The eradication rate of CAZ-Lip (*n* = 5/group) **(F)**.

As shown in Figure 1A, all liposomes had a uniform particle size of 100 nm with typical bilayer vesicle structures, and the loading of berberine did not affect the size and structure of liposomes. The particle size and zeta potential of the free liposome and the BH-Lip did not change significantly over 21 days, indicating the good stability of the nanoparticles (Supplementary Figure S1; Supplementary Table S1). The inhibition rate for planktonic *S. aureus* was almost 100% when the concentration of free berberine was 50 μg/mL, while the inhibition rate of berberine liposomes against *S. aureus* was significantly higher than that of free berberine at lower concentration (Figure 1B). The biofilm eradication rates of the liposome-encapsulated berberine were higher than that of the free drug, which demonstrated that the liposomes as carriers fully exploited their ability to penetrate the biofilm, allowing berberine to be more fully targeted to bacteria inside the biofilm.

The biofilm eradication rate of 50 μg/mL BH-Lip was about 60%, which did not decrease significantly with the decrease in concentration. In contrast, the biofilm eradication rate of free berberine solution was significantly lower than that of liposomes, which was only about half of that of liposomes (Figure 1C). Ciprofloxacin and cephradine, two widely used antibiotics, did not remove biofilms, especially at high concentrations, indicating the resistance of biofilms to antibiotics, which is consistent with previous reports. However, both of them showed efficient biofilm removal ability when encapsulated in liposomes (Figures 1D–F). Considering the biofilm eradication stability of berberine and its liposomes, we selected berberine as the model for *in vitro* and *in vivo* experiments in subsequent experiments.

### 3.2 *In vitro* experiments showed that *in-situ* thermosensitive hydrogel loaded with liposome could inhibit *S. aureus* and its biofilm

**FIGURE 2:**
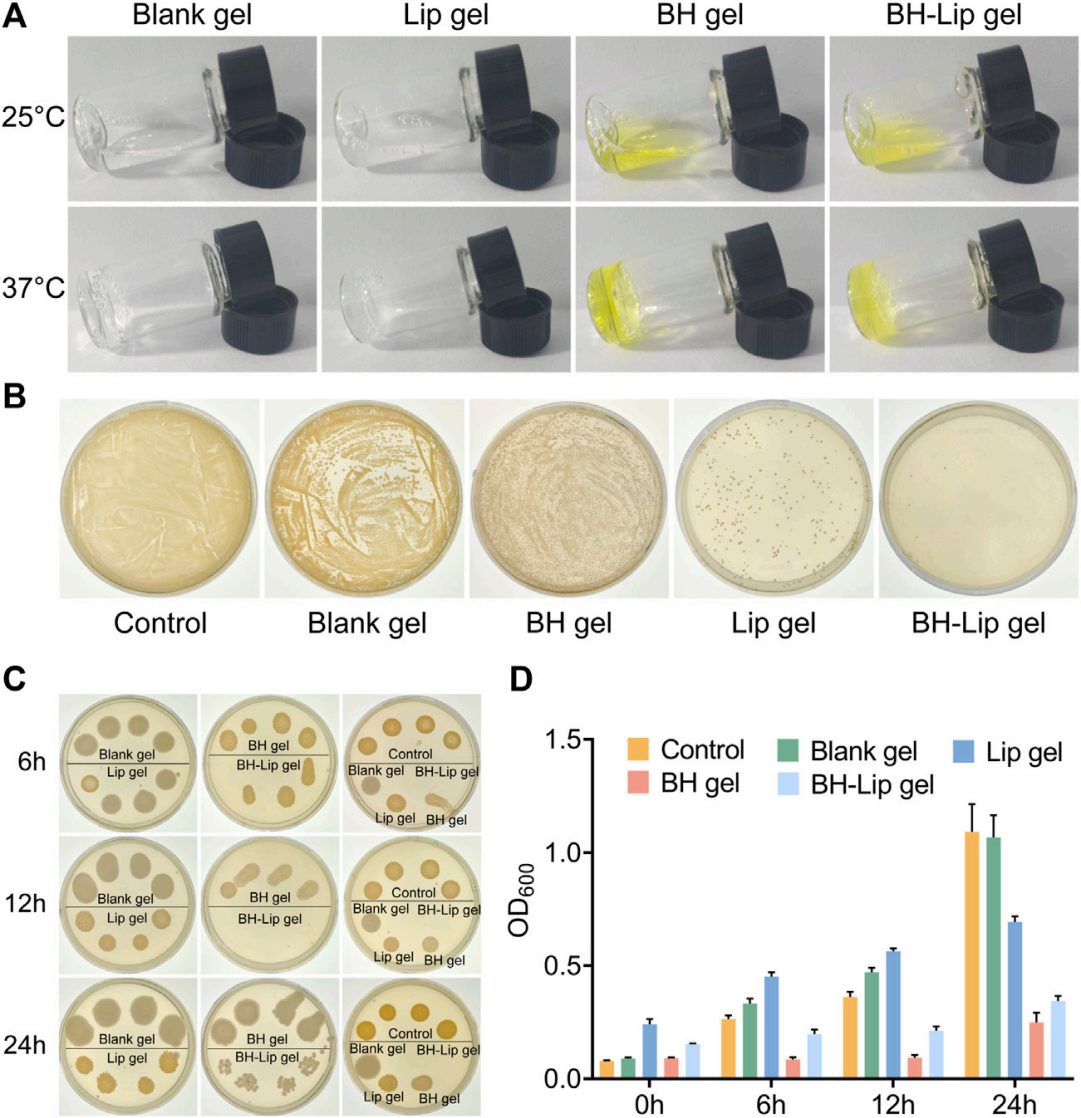
Four temperature-sensitive gels at phase change temperature **(A)**. The inhibition effect of four gels on *S. aureus* was verified by dilution coated plate method (diluted 10^3^ times, *n* = 3/group) **(B)**. Antimicrobial effect of four gels against *S. aureus* after incubation of 6, 12, and 24 h **(C)**. Absorbance of the four gels after incubation with *S. aureus* at different periods (*n* = 6/group) **(D)**.

As shown in Figure 2A, all four temperature-sensitive gels are liquid with the good flow at room temperature. After being heated in a 37°C water bath they rapidly transform into semi-solid gels. After repeated testing, all four gels have good gel-forming properties and the process of gel formation typically takes less than 1 min, allowing them gelling *in situ*. In addition, the gel-loaded drugs were released slowly *in vitro* and stabilized at 36 h. Finally, the cumulative release rate was about 70% (Supplementary Figure S2). Poloxamer, the material used to form the gels, is a (PEO-PPO-PEO) triblock copolymer consisting of a hydrophobic chain poly (propylene oxide) (PPO) and a hydrophilic chain poly (ethylene oxide) (PEO) (Bodratti and Alexandridis, 2018; Zarrintaj et al., 2020). When the temperature rises to the phase transition temperature of the system, Poloxamer can self-assemble into a micelle structure through hydrophobic interactions between PPO and PEO, then the micelles build up on each other, exhibiting a reversible sol-gel transition and form a network structure (Chatterjee et al., 2019; Russo and Villa, 2019).

The four gels differed significantly in their inhibition of *S. aureus*, with the BH-Lip gel showing the best performance. The blank gel showed almost no inhibition effect, and the Lip gel had a slightly stronger inhibition effect than the BH gel (Figure 2B). We then examined the inhibition effect exerted by the four gels at different times. The results showed that the BH-Lip gel was still the most effective regardless of the period and showed the strongest efficacy at 12 h. BH gel is unable to target bacteria as stably as BH-Lip gel, which also highlights the characteristics of liposomes as carriers to improve the effectiveness of antibacterial drugs (Figures 2C, D).

### 3.3 *In-situ* thermosensitive hydrogel loaded with liposome have anti-inflammatory and antibacterial effects *in vivo*

**FIGURE 3:**
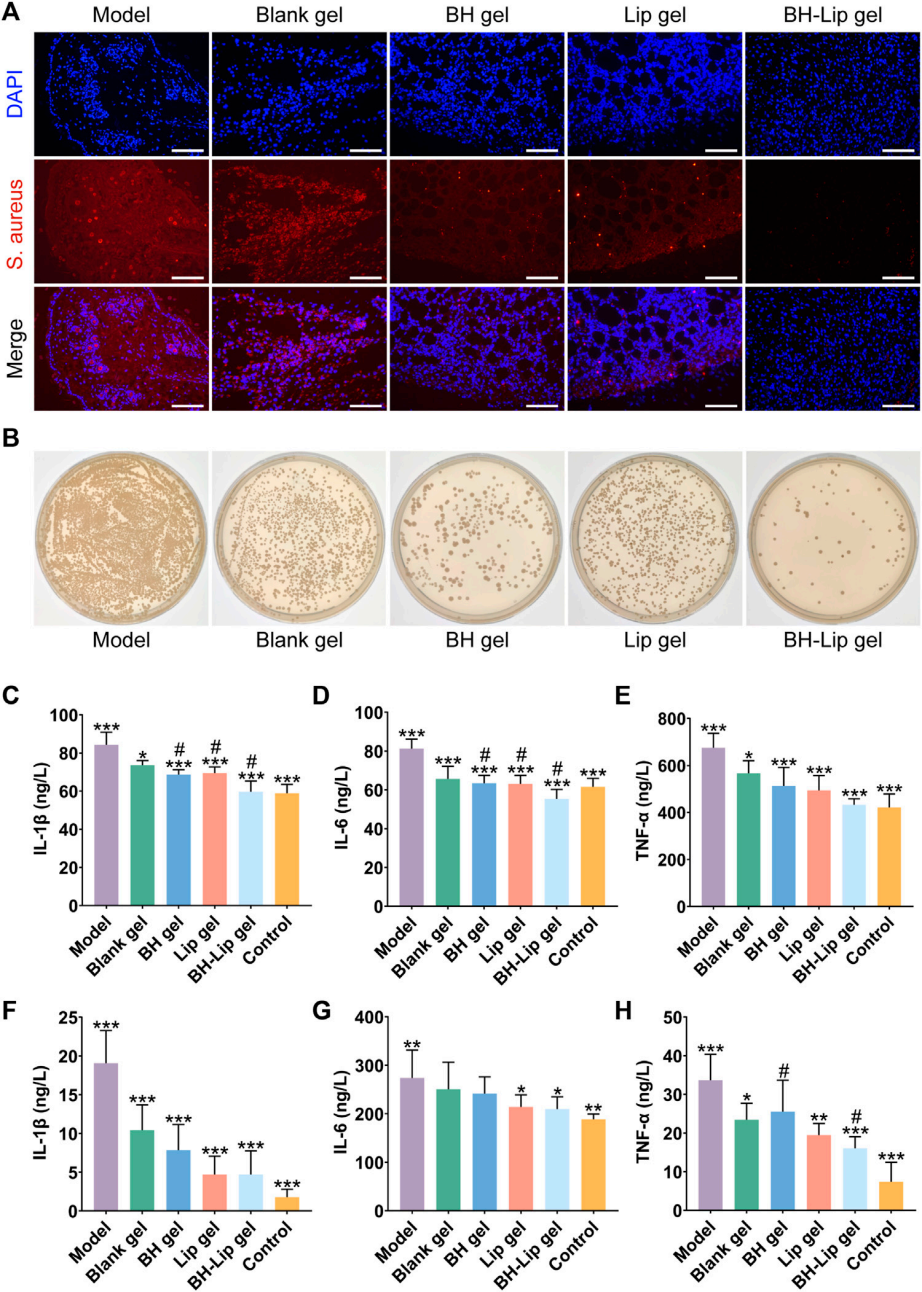
FISH analysis of wound tissue from different groups of mice (Scale bar = 50 µm). The wound tissue of *S. aureus* was labeled by a specific ribosomal RNA (rRNA) FISH probe (Red). The nuclear region of the bacteria was stained (blue) after DAPI (4′,6-diamidino-2-phenylindole) counterstaining **(A)**. Comparison of the number of viable bacteria in the wound tissues after treatment with the four different gels by dilution coating plate method (diluted 10^3^ times, *n* = 3/group) **(B)**. Quantitative analysis of IL-1β, IL-6, and TNF-α levels in wound tissue on day 5 after treatment (***p < 0.01, *p < 0.05 when compared with the model group, ^**#**^ *p* < 0.05 when compared with the BH-Lip gel group, *n* = 6/group) **(C,D,E)**. Quantitative analysis of IL-1β, IL-6, and TNF-α levels in wound tissue on day 14 after treatment (***p, **p < 0.01, *p < 0.05 when compared with the model group, ^**#**^ *p* < 0.05 when compared with the BH-Lip gel group, *n* = 6/group) **(F,G,H)**.

Encouraged by the *in vitro* efficacy, we further conducted *in vivo* trials for the treatment of wound infections, and explored the effect of BH-Lip temperature-sensitive gel on the removal of biofilm from infected wounds. We used fluorescent *in situ* hybridization (FISH) to locate the bacteria and their biofilm on the wound utilizing a gene probe. This technique has the advantage of rapid identification of pathogens and more visualization of the results (Frickmann et al., 2017; Prudent and Raoult, 2019; Wu et al., 2019). As shown in Figure 3A, fluorescence images of *S. aureus* were obtained by FISH of the wound tissue after 5 days of administration (the red fluorescence shows hybridization of specific probes targeting *S. aureus* rRNA and blue fluorescence represents the nucleus). The model group showed the most intense red fluorescence signal, which was concentrated in a regional distribution, highly similar to the high density of the biofilm and the clustered nature of the contact surface. Therefore, it could be judged that the model of the biofilm of *S. aureus* colonizing the wound was successfully constructed.

The red fluorescence intensity of the blank gel group was slightly lower than that of the model group but much greater than that of the remaining three groups, indicating that the blank gel alone was unable to exert an antibacterial effect, which is also consistent with the results of previous *in vitro* experiments. The red fluorescence intensity of the BH-gel, Lip-gel, and BH-Lip gel groups decreased in turn, and the difference between the BH-gel and Lip-gel groups was not significant, while the BH-Lip gel group had a clear difference from the other groups and the red fluorescence signal was already almost invisible, indicating that BH-Lip gel successfully exerted its drug effect *in vivo* after 5 days of treatment. We further investigated the antimicrobial effect of the gel on the wound using the dilution coating plate method (Figure 3B). Interestingly, the BH gel group had fewer colonies than the Lip gel group, which is different from the previous results of the *in vitro* dilution coating plate method. There is no doubt that the BH-Lip gel group had the lowest number of colonies compared to the other groups and showed the best results.

The expression levels of three inflammatory factors, IL-Iβ, IL-6, and TNF-α, were considerably lower in the BH gel, Lip gel, and BH-Lip gel groups than in the model group 5 days after administration. The level of the three inflammatory factors in the BH-Lip gel group, which had the strongest anti-inflammatory effect, was reduced to a level close to that of the blank group, and its expression levels of IL-1β and IL-6 were significantly different from those of the BH gel and Lip gel groups. The difference between the blank gel group and the model group was not as prominent as in the other groups, indicating that blank gel had a smaller effect on inflammation abatement (Figures 3C–E).

We also measured the expression levels of the three inflammatory factors in tissues 14 days after administration (Figures 3F–H), and found that the expression levels of IL-1β and TNF-α decreased in all groups compared to the pre-treatment period of days 0–5. Overall, the organism was in a continuous dynamic balance of inflammation levels during the transition from the early to the late phase of treatment. However, the difference was that the level of elimination of the three inflammatory factors was generally stronger in the Lip gel group than in the BH-gel group during the late phase of treatment, which was a novel finding.

### 3.4 BH-lip hydrogel promoted wound healing by eliminating inflammation and accelerating angiogenesis

**FIGURE 4:**
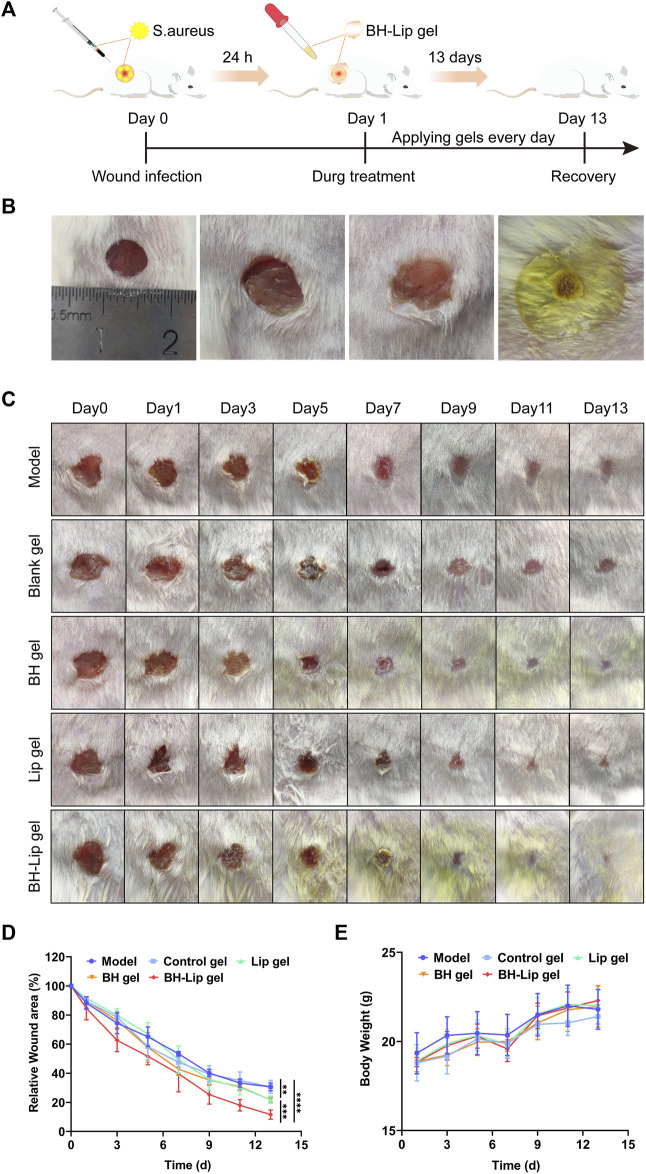
Experimental schematic of BH-Lip gel-mediated enhanced wound healing effect in the *S. aureus* infection model **(A)**. Comparison of the appearance of the wound before and after infection, molding day 0 (left), molding day 1 (middle two pictures), and gel forming rapidly on the wound surface (right) **(B)**. Wound healing after treatment in different groups on days 0, 1, 3, 5, 7, 9, 11, and 13 **(C)**. Quantification of wound healing area (****p < 0.01 when comparing the BH-Lip gel group with the model group, ***p < 0.01 when comparing the Lip gel group and the BH gel group with the BH-Lip group, **p < 0.01 when comparing the Lip gel group and the BH gel group with the model group, *n* = 5/group) **(D)**. Weight of mice over 14 days (*n* = 5/group) **(E)**.

To determine whether BH-Lip gel can accelerate healing of infected wounds, we established a full-layer skin resection model in mice and continued treatment for 13 days (Figure 4A). As shown in Figure 4B, the wound was regularly rounded (approximately 6 mm in diameter) on day 0 of molding and emerged with golden yellow bacterial aggregates that were highly adherent after 24 h of infection. After 13 days of treatment, the wound healing in different groups is shown in Figure 4C. It can be seen that the BH-Lip gel group had the best healing effect on day 13, while the BH gel group and Lip gel group also had some effect on wound healing. After quantification of the wound area by ImageJ software (Figure 4D), it was easy to see that the three gel groups with significant healing (Lip gel, BH gel, and BH-Lip gel) had a lower wound shrinkage rate on day 5 than on the other days. When analyzed together with the three inflammatory factors measured on the fifth day of treatment, it was found that the fifth day was the time when the inflammation subsided. Thus, the degree of remission of inflammatory factors was proportional to the degree of wound healing and the drug-laden gel further promoted subsequent wound healing by reducing the inflammatory factors on the wound. During these 13 days, the trend in body weight change was normal in all mice **(** Figure 4E).

**FIGURE 5:**
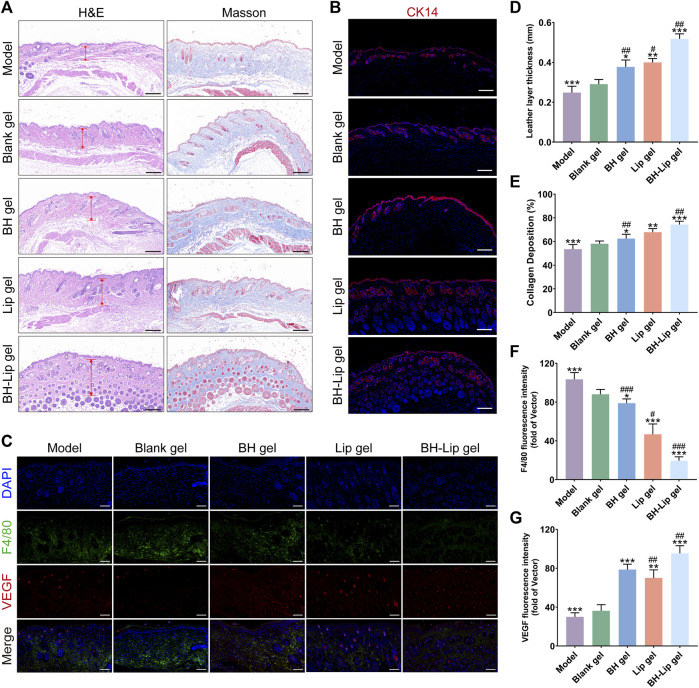
H&E and Masson staining of wound tissues of different groups (Scale bar = 200 µm) **(A)**. Immunofluorescence staining of CK14 (red) for the wounds at days 14 (Scale bar = 200 µm), cells were stained with CK14 and nuclei were re-stained with blue DAPI **(B)**. Immunofluorescence staining of F4/80 (green) and VEGF (red) for the wounds at day 14 (Scale bar = 100 µm) **(C)**. Quantification of the length of the new leather layer of the wound (***p, **p < 0.01, *p < 0.05 when compared with the model group, ^**##**^ *p* < 0.01, ^**#**^ *p* < 0.05 when compared with the BH-Lip gel group, *n* = 6/group) **(D)**. Maturity of collagen fibers in different groups (***p, **p < 0.01, *p < 0.05 when compared with the model group, ^**##**^ *p* < 0.01 when compared with the BH-Lip gel group, *n* = 6/group) **(E)**. Quantification of F4/80 fluorescence intensity (***p < 0.01, *p < 0.05 when compared with the model group, ^**###**^ *p* < 0.01, ^**#**^ *p* < 0.05 when compared with the BH-Lip gel group, n = 6/group) **(F)**. Quantification of VEGF fluorescence intensity (***p, **p < 0.01 when compared with the model group, ^**##**^ *p* < 0.01 when compared with the BH-Lip gel group, *n* = 6/group) **(G)**.

As shown in Figure 5A, images of cross-sections of wound tissue from different groups after H&E staining and Masson staining were used to further assess wound healing. The BH gel group, Lip gel group, and BH-Lip gel group were covered with a continuous and thick epidermal layer. The regenerated dermis (red arrows) was significantly thicker in these three groups relative to the model and blank gel groups, with the BH-Lip gel group first and the Lip gel group slightly thicker than the BH gel group (Figure 5D). The Masson staining reflects the degree of collagen deposition during the wound healing phase, and the intensity of the blue staining represents the number and maturity of collagen fibers. The BH-Lip gel group had the most closely packed collagen fibers, the darkest blue color, and far more new hair follicles than the other groups, followed by the Lip group, while the BH gel group was inferior to the above two groups. These pathological results were also consistent with the trend of the H&E staining results (Figure 5E), demonstrating that BH-Lip gel can promote wound healing and tissue regeneration.

It has been reported that the rate of wound healing is closely related to the degree of re-epithelialization (Guan et al., 2021). Keratin is the structural protein of epidermal cells and they form extremely fine microfilaments that build an extensive intracellular meshwork. Keratin promotes keratin-forming cells and maintains their integrity in the epithelium, with epidermal cells expressing different keratins depending on the degree of differentiation (Pan et al., 2013; Konop et al., 2021; Qiang et al., 2021). In contrast, cytokeratin 14 (CK14), a type of keratin, is a marker for basal keratin-forming cells and is essential for wound re-epithelialization (Cheng et al., 2020). As shown in Figure 5B, in the BH-Lip gel group, both the new hair follicle and the wound gap were filled with CK14-forming cells (red fluorescence), and keratinocytes continued to migrate from the dermis to the epidermis and formed a thick spine layer in the stacked epidermis, such a phenomenon was also present in the Lip gel group as well as the BH gel group. However, in the blank gel group and the model group, the expression of CK14 was relatively low, the cell proliferation rate was not as fast as the other three groups, and the re-epithelialization was not mature enough, which fully indicates that the re-epithelialization in the BH-Lip gel group was far ahead, and the drug effectively played the role of promoting the proliferation and migration of keratinocytes.

F4/80 is a cell surface glycoprotein, the expression of F4/80 changes markedly during the maturation and activation of macrophages (Biguetti et al., 2018). From another perspective, the level of F4/80 expression is to some extent proportional to the level of inflammation in the tissue (Zhou et al., 2018; Yang et al., 2020). Compared to the Lip gel and BH-Lip groups, intense green fluorescence was detected in the model and blank gel groups, indicating that there was still excessive inflammation in the tissues, which was not conducive to wound healing. the fluorescence intensity of F4/80 in the Lip gel group was significantly different from that of the BH-Lip gel group and much greater than that of the BH gel group, and we judge that it could be that drugs induce an inflammatory response in the organism and that liposomes, as affinity carriers, have a similar composition to that of cell membranes, thus well avoiding the irritation of drugs (Figures 5C, F). VEGF is a key factor in the formation of new blood vessels, and continuous treatment with BH-Lip gel promoted the secretion of VEGF (red fluorescence) in the wound, which in the case of a high number of new blood vessels, led to the proliferation of granulation tissue in the wound and the ability of the tissue to fibrosis was enhanced (Yao et al., 2020). In conclusion, immunofluorescence staining analysis showed that BH-Lip gel promoted healing by increasing the rate of wound re-epithelialization and angiogenesis (Figures 5C, G).

## 4 Discussion

There are many treatments for biofilm-related wounds, but scientists and medical professionals have yet to find the most effective and efficient combination of treatments. Current treatments fall into three main categories: mechanical debridement, antibiotics, and anti-biofilm/biofilm-destroying agents such as QS blockers. However, none of the three categories on its own will provide adequate treatment. Mechanical debridement, such as surgery, often cannot completely remove biofilms because the EPS matrix penetrates deep into tissue structures. Any residue will quickly restore the biofilm. In addition, debridement needs to be carried out continuously, and only 25% of the wounds with irregular debridement can heal, while 83% of the wounds with weekly debridement can heal. Antibiotic treatment alone is also not sufficient, because biofilms have many resistance mechanisms.

In this work, we have developed a multilayer liposome with a particle size of approximately 100 nm that can be loaded with berberine hydrochloride, ceftazidime, and ciprofloxacin, antibiotics that target delivery of drugs to penetrate the bacterial biofilm to fight the bacteria within the membrane. For better application in infected wounds, we have taken advantage of the temperature-sensitive properties of the Poloxamer series of gels to combine drug-containing liposomes with the gels to allow the drug to reach the biofilm deep within the wound and thus take full effect.

On the one hand, *in vitro* experiments have shown that these antibiotic-containing liposomes are not only effective against Gram-positive bacteria (*S. aureus*), but also against Gram-negative bacteria (*Pseudomonas aeruginosa*), which confirms the advantages of liposomes as drug carriers for targeted delivery. In addition, we found that the absorbance of the BH gels after co-incubation with the bacteria did not correspond to the inhibition effect on agar plates, which we speculate may be related to the environment (liquid or solid) in which the gels release the drug and need to be further investigated. We also found that Lip-gel showed a stronger inhibition effect than BH-gel *in vitro* and that the antibacterial and anti-inflammatory effects exerted in the *in vivo* experiments were significant, and that the inhibition effect exerted by the blank liposomes is not negligible and has some value for future research.

On the other hand, the *in vivo* results showed that BH-Lip gel not only fights the biofilm on the wound but also plays a role in promoting wound healing. In general, wound healing goes through four stages: coagulation, inflammatory response, cell proliferation, and tissue remodeling, and the regulation of inflammation is crucial in the wound healing process. BH-Lip gel is effective in shortening the inflammatory phase of the wound, accelerating angiogenesis and collagen deposition after the inflammation has subsided (after the 5th day of administration), thus promoting wound healing. One of the crucial mechanisms of wound treatment is the regulation of the macrophage M1/M2 balance. Macrophages in trauma have both phagocytic and secretory functions. During the period of wound infection, M1-type macrophages can phagocytose and remove exogenous foreign bodies and necrotic cells, while in the stage of trauma recovery, M2-type macrophages can regulate wound repair by releasing various cytokines (Kobashi et al., 2020; Tang et al., 2023). The anti-inflammatory effect of BH-Lip gel could be interpreted as the pharmacological activity of berberine, which was able to regulate inflammation by inducing macrophages to differentiate from M1 to M2 type, and reducing the production of TNF-α, IL-6 and then improve the local inflammatory environment and promote lipid clearance and metabolism (Ehteshamfar et al., 2020).

## 5 Conclusion

Colonization of wounds by bacterial biofilm is one of the major causes of obstruction of infected wound healing. There are various clinical treatments for wound infections, such as surgical debridement (Wormald et al., 2020), ultrasound therapy (Lyu et al., 2021), negative pressure therapy (Iheozor-Ejiofor et al., 2018), the use of appropriate dressings and the use of antibiotics (Hu et al., 2022; Zhou et al., 2022). However, due to the misuse of antibiotics, a large number of common bacteria have developed resistance and the biofilm has a complex internal structure and multiple mechanisms of resistance to external stimuli, making it highly resistant to both immune responses and antimicrobial drugs. Because biofilms are difficult to remove completely, new therapeutic strategies to combat biofilms are now urgently needed.

Berberine, a bright yellow isoquinoline alkaloid existing in a variety of natural plants, has been widely studied and reported for its antibacterial effects. Berberine has antibacterial mechanisms such as inhibition of bacterial division, disruption of bacterial structure and interference with bacterial metabolism (Bhatia et al., 2021). However, its clinical application is limited by its poor solubility properties and short residence time in tissues (Wang et al., 2017). In this study, an *in-situ* temperature-sensitive hydrogel loaded with berberine liposomes was developed and applied to the treatment of infected wounds in mice, which led to a slower release of the drug and enhanced drug retention time in the body. The results showed that berberine liposome and its hydrogel could significantly inhibit *S. aureus* and eradicate biofilm *in vitro*. When the BH-Lip comes into contact with the biofilm, it can effectively penetrate the biofilm structure and release berberine directly into the biofilm-embedded cells. This effect is related to the high content of cholesterol in liposomes which attracts bacterial pore-forming toxins and the release of drugs resulting from pore-forming. This function of liposomes can be attributed to the good fusion of liposomes on bacterial biofilms. α-Hemolysin, which is secreted by *S. aureus*, forms small pores that primarily affect the intracellular ion balance. α-Hemolysin binding depends on the presence of certain lipid and/or liquid-ordered lipid microdomains within the liposomes, which is consistent with the composition of host cell plasmalemma (Pornpattananangkul et al., 2011; Jiang et al., 2021). Therefore, liposomes can act as decoys to isolate bacterial toxins and thus achieve the purpose of protecting host cells. *In vivo* experiments showed that the thermosensitive hydrogels containing berberine liposome could significantly remove bacterial biofilms from wounds, inhibit inflammatory factors and promote wound recovery. In conclusion, current research confirmed that hydrogel-mediated BH-Lip could be considered as an effective nanomaterial and that BH-lip gel promoted wound healing by eliminating inflammation and accelerating angiogenesis. This temperature-sensitive hydrogel we have developed can be applied to biomedical applications, including targeted drug delivery, tissue adhesion, and tissue engineering. Our study provides an effective management method for biofilm-associated wound infections and is expected to play a greater role in clinical practice.

## Data Availability

The raw data supporting the conclusion of this article will be made available by the authors, without undue reservation.

## Abbreviations

BH-Lip, Berberine liposome; CAZ-Lip, Ceftazidime liposome; CIP-Lip, Ciprofloxacin liposome; CK14, cytokeratin 14; EPS, Extracellular polymeric substances; FISH, Fluorescent *in situ* hybridization; VEGF, Vascular endothelial growth factor.
