# Supplementary material for: In situ gelling hydrogel loaded with berberine liposome for the treatment of biofilm-infected wounds
Source: Front Bioeng Biotechnol. 2023 May 31;11:1189010. doi: 10.3389/fbioe.2023.1189010 (PMC10266532; doi:10.3389/fbioe.2023.1189010)
Supplement: Supplementary file 1 [file DataSheet1.PDF]

Supplementary Material

In Situ Gelling Hydrogel Loaded with Berberine Liposome for the Treatment of Biofilm-Infected Wounds

Sipan Li [a], Yongan Wang [a], Siting Wang [a], Jianjun Xie [a], Tingming Fu [a*], Shaoguang Li [b*]

*** Correspondence:**
Tingming Fu*（futm@njucm.edu.cn）
Shaoguang Li*(drlishaoguang@163.com)

1 Supplementary Figures and Tables

1.1 Supplementary Figures

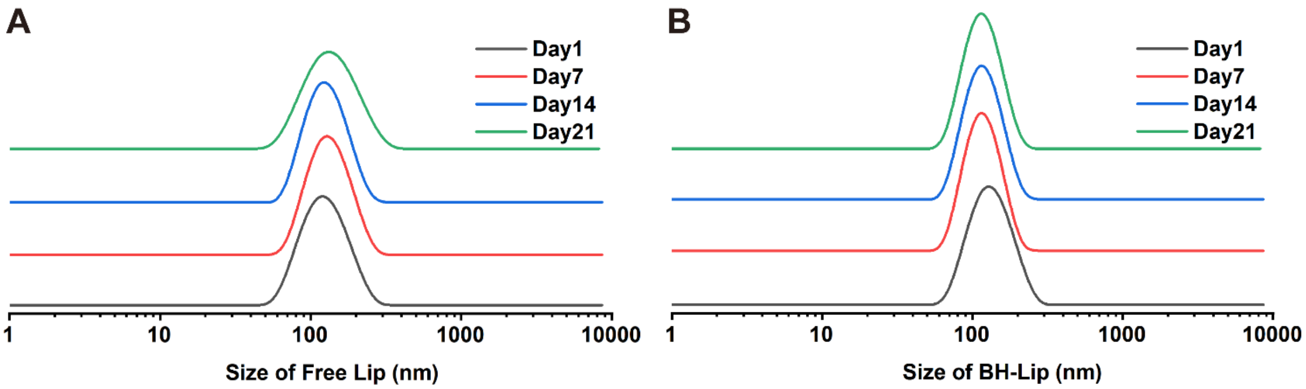

Supplementary Figure 1. Particle size of Free Lip over 21 days **(A)**. Particle size of BH-Lip over 21 days **(B)**.

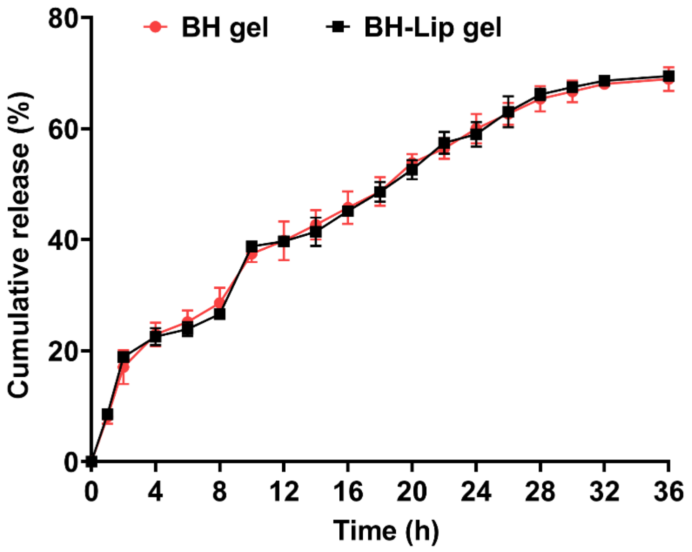

Supplementary Figure 2. In vitro release of BH gel and BH-Lip gel (n=3/group).

1.2 Supplementary Tables

Supplementary Table 1. Zeta potential of Free Lip and BH-Lip (mV, Mean±SD)

	Day 1	Day 7	Day 14	Day 21
Free Lip	-8.26±0.41	-10.37±0.25	-7.45±0.31	-7.26±0.30
BH-Lip	-4.39±0.82	-5.31±0.46	-8.95±1.06	-4.81±0.40
